# Experience and perceptions among rheumatoid arthritis patients following a telehealth-delivered dietary intervention: a qualitative study

**DOI:** 10.1007/s00296-023-05382-7

**Published:** 2023-07-07

**Authors:** Tala Raad, Anne Griffin, Elena S. George, Louise Larkin, Alexander Fraser, Norelee Kennedy, Audrey Tierney

**Affiliations:** 1 Discipline of Dietetics, Faculty of Education and Health Sciences and Health Implementation Science and Technology Cluster, School of Allied Health, Health Research Institute, University of Limerick, Limerick, V94 T9PX Ireland; 2 Institute for Physical Activity and Nutrition (IPAN), School of Exercise and Nutrition Sciences, Deakin University, Victoria, VIC 3220 Australia; 3 Discipline of Physiotherapy, School of Allied Health, Faculty of Education and Health Sciences, Implementation Science and Technology Centre, Health Research Institute, University of Limerick, Limerick, V94 T9PX Ireland; 4 Department of Rheumatology, University Hospital Limerick, Limerick, V94 T9PX Ireland; 5 Faculty of Education and Health Sciences, Graduate Entry Medical School, University of Limerick, Limerick, V94 T9PX Ireland; 6 Faculty of Science and Engineering, School of Allied Health, Human Services and Sport, La Trobe University, Melbourne, VIC 3086 Australia

**Keywords:** Rheumatoid arthritis, Mediterranean diet, telehealth, Qualitative, Focus group, Dietary intervention

## Abstract

The effects of dietary modifications have been assessed in people living with rheumatoid arthritis (RA) with consistent benefits reported from clinical trials. However, the lived experience of making and sustaining positive dietary changes for people with RA remains unknown. The aim of this qualitative study was to explore the experiences of adults with RA and their perceptions of a 12-week telehealth-delivered dietary intervention and to assess the acceptability of the programme. Qualitative data was collected via four online focus groups with participants who had just completed a 12-week dietary intervention programme delivered through telehealth methods. Thematic analysis was used to code and summarize the identified key themes. Twenty-one adults with RA (47.5 ± 12.3 years, 90.5% females) were included in this qualitative study. Overarching themes included: (a) motivation to join the programme, (b) benefits of the programme, (c) factors influencing adherence to dietary prescription, and (d) advantages and disadvantages of telehealth. The study demonstrated that a dietary intervention delivered through telehealth methods by a Registered Dietitian (RD) appears to be well-accepted and may be used to complement face-to-face care for people with RA. The identified factors influencing the adoption of a healthier eating pattern will aid in the development of future dietary interventions for a RA population.

## Introduction

Rheumatoid arthritis (RA) is a chronic inflammatory disease affecting approximately 0.5–1% of the adult population worldwide [1]. More than 40,000 people are currently living with the condition in Ireland [2]. Three quarters of those diagnosed with RA are female and the average age for diagnosis is between 30 and 50 years [3]. RA is a common and often debilitating joint disease with physical, social and psychological consequences thereby, severely impacting an individual’s quality of life [4]. Despite advancements in targeted biologic and pharmacologic treatments, there is still no cure for RA [5]. People with RA are at an increased risk of cardiovascular disease (CVD) with cardiovascular mortality accounting for over half of all deaths in RA [6]. Therefore, strategies to reduce cardiovascular risk are considered an important part of RA management. The influence of positive dietary and lifestyle changes has been well documented in CVD [7–9]. Similarly, improvements in symptoms, disease activity, physical function, and quality of life have been reported from lifestyle intervention studies in RA [10–12].

There is a growing body of evidence supporting the role of diet for improving health outcomes in RA [13]. The anti-inflammatory nature and cardiovascular benefits of the Mediterranean diet (MedDiet) have generated interest in this dietary pattern for the management of RA [11, 14]. Most clinical trials assessing the effectiveness of a MedDiet have been conducted in Mediterranean regions [15]. However, given the proven benefits of the MedDiet in the prevention and treatment of chronic diseases, it is worthwhile assessing this dietary pattern in non-Mediterranean countries where rates of such diseases are high. The Mediterranean Dietary Intervention for Adults with Rheumatoid Arthritis (MEDRA) is a 12 week, two-arm parallel group, randomised controlled trial comparing the effects of a MedDiet versus adhering to the Irish Healthy Eating Guidelines (HEG) on physical function and quality of life in adults with RA in Ireland [16]. Participants in the MedDiet group adhered to the principles of a traditional Cretan MedDiet principles and were advised to consume 60–80 ml of Extra Virgin Olive Oil (EVOO), at least two servings of vegetables and three servings of fruits daily. Participants were also instructed to include fish at least 3 times per week and a handful of raw unsalted nuts every other day. Participants who were allocated to the HEG group were instructed to follow the Irish Healthy Eating Guidelines published by the Department of Health in December 2016. In this intervention arm, participants were requested to consume 5–7 servings of fruits and vegetables, 3–5 servings of wholegrain cereals, 3 servings of low-fat dairy, 2 servings of lean meat per day. Participants were asked to limit the intake of high fat, sugar, salt food and drinks to once or twice per week and to use fats, spreads, and oil in very small amounts. Both diet groups attended three video teleconsultations (baseline, 6, and 12 weeks) and two follow-up phone calls at weeks 3 and 9. The dietary interventions comprised personalised dietary advice and nutrition counselling including nutrition education and goal setting from the RD to help improve dietary quality and achieve adherence to the prescribed diet.

While it is crucial to assess effectiveness of interventions, it is also imperative to understand the experiences and perceptions of participants taking part in these studies through a qualitative exploration [17]. Synthesising quantitative and qualitative evidence provides a deeper and more holistic perspective into a specific population’s behaviours [18]. Moreover, qualitative approaches are critical for implementation and are highly valuable for understanding complex social behaviours such as engaging in dietary studies and making sustained changes to dietary patterns [19]. Voshaar et al. emphasises the importance of a patient-centred approach in RA care; the authors indicate that involving participants as individuals, with unique needs, perceptions and preferences, has relevant impact on treatment outcomes such as safety and effectiveness [20].

In recent years, a number of qualitative studies have indicated that behavioural change interventions delivered through telehealth methods are positively perceived by people with RA [21, 22]. However, to the authors knowledge, there is currently no study evaluating the views of people with RA on dietary intervention programmes. In addition, no studies have been conducted to determine the barriers and enablers to making dietary changes or improving dietary quality in this population. To address this gap in the literature and to support the objective findings of the MEDRA trial, this qualitative study aims to explore participants’ experiences following the completion of the MEDRA trial and to determine key factors influencing the adoption of a new dietary pattern. Since the COVID-19 pandemic required a rapid adoption of telehealth to reduce the likelihood of virus transmission [23], this qualitative study also explores participants perspectives on the use of telehealth as a mode of service delivery. Developing a thorough understanding of the perceived barriers towards adopting a new dietary pattern and ways to overcome them is crucial for informing the scale up of effective dietary interventions with sustainable dietary changes as well as developing dietary guidelines for a RA population.

## Methods

### The MEDRA trial

The MEDRA trial is a 12-week dietary intervention delivered through telehealth methods. The trial comprised three video teleconsultations with a Registered Dietitian (RD) at weeks 0, 6 and 12 and two follow-up phone calls at weeks 3, and 9. The MEDRA trial protocol is published elsewhere [16]. The trial aimed to assess the effectiveness of a MedDiet compared to the HEG in terms of improvements in physical function and quality of life in adults with RA residing in Ireland.

In response to social media advertisement and posters display at University Hospital Limerick outpatient rheumatology clinics, participants were enrolled to the trial by the study RD and randomised into either MedDiet (*n* = 22) or HEG group (*n* = 22). Participants in both diet groups attended a baseline nutrition consultation session whereby they were informed of their group allocation. Participants in the MedDiet group were advised to follow the principles of the traditional Cretan Mediterranean diet that were tailored to an Irish population [16]. Participants in the HEG group were advised to follow the Healthy Eating Guidelines currently recommended in Ireland [24]. Participants in both groups received resources to inform them of the dietary guidelines and facilitate adherence to the prescribed diet.

### Study design

This qualitative study employed the use of semi-structured focus groups and was informed by the Consolidated criteria for reporting qualitative research (COREQ) checklist [25]. The chosen data collection method of a focus group allows participants to assess their responses and opinions against those of others in the group therefore providing in-depth information [26].

### Participants and sampling

All participants in the MEDRA trial were adults (age ≥ 18 years) with a definite diagnosis of RA and full access to the internet or a mobile phone. Individuals who were pregnant or breastfeeding were excluded. Full details of the inclusion/exclusion criteria have been previously reported in full elsewhere [16]. Following the 12-week dietary intervention period, all 40 participants who completed the MEDRA trial (January 2021–May 2021) were invited by email from the RD to take part in focus groups to share their experience of the programme retrospectively.

### Ethics

Ethics approval for this qualitative study was obtained within the application for the overarching MEDRA trial which has been approved by the Education and Health Sciences Research Ethics Committee at the University of Limerick (2020_09_05_EHS) and by the Health Service Executive Mid-Western Regional Hospital Research Ethics Committee (REC Ref 103/19). All participants signed an informed consent form assuring anonymity, confidentiality, and freedom to withdraw from the study. Participants were not provided with financial incentives.

### Focus groups

Data collection for the present study was initiated in May 2021. All focus groups were conducted virtually through Microsoft Teams. Focus groups were audio recorded and recordings were transcribed verbatim (TR) while maintaining anonymity and confidentiality of participants. All focus groups were facilitated by the same moderator (AT) who had no previous contact with participants within the trial. A notetaker (TR) was also present to record key points and observe emerging concepts and ideas. The moderator followed a semi-structured questioning guide based on the study’s aims and adapted questions from similar qualitative studies exploring participants’ experiences following a lifestyle intervention [27]. The guide included questions regarding participants’ reasons for taking part in the MEDRA trial, perspectives, and experiences with the dietary intervention programme, making and sustaining dietary changes, and the challenges related to the telehealth delivery method. The focus group questions can be found in Table 1.

**Table 1:** Focus groups questioning guide

1	Why did you decide to join this programme?
2	What did you think of this programme? Was it what you expected?
3	Describe your overall experience of being part of this study
4	Did you achieve the results you hoped for OR do you feel that your overall health improved while following the prescribed diet?
5	Have you ever heard of about a MedDiet/HEG before this study?
6	What do you believe were the advantages of following a MedDiet/HEG?
7	What were the positive effects of the MedDiet/HEG on your symptoms (e.g., pain, stiffness, energy levels.)?
8	Did you feel satisfied while following a MedDiet/HEG? (Or did you feel hungry and deprived?)
9	Was the prescribed diet hard to adhere to?
10	What do you believe were the disadvantages to following a MedDiet/HEG?
11	What factors or circumstances would make it difficult or impossible for you to following a healthy diet?
12	What factors or circumstances would enable you to follow a healthy diet?
13	In your opinion, how did the pandemic affect your experience?
14	How did the restrictions impact your experience? (e.g., physical activity)
15	How did you feel about the intervention being conducted through telehealth methods? Did you feel comfortable during the teleconsultations?
16	How do you rate the quality of service delivered through telehealth methods when compared to the quality traditional care?
17	What are specific benefits to using telehealth?
18	What are potential challenges to using telehealth?
19	Do you believe you will continue to consume the same diet?
20	Would you recommend this diet to someone living with rheumatoid arthritis?

In order to demonstrate rigor in this study, we ensured data saturation for adequate sample size through the identification of adequate information for study replication and when no additional new information was obtained [28]. A qualitative descriptive (QD) approach was employed in this study. This approach, informed by naturalistic inquiry, offers a practical way for understanding the experiences of participants from a subjective perspective [29]. QD is a popular approach used for gaining insight into a specific topic from the perspectives of participants. The approach has been recommended for use in healthcare contexts because it provides insight into patient’s and clinicians’ experiences and perspectives in relation to a specific topic [30].

### Data analysis

The analytical strategy aimed to understand people’s subjective views on undertaking a dietary intervention programme delivered by a RD through telehealth methods. The end goal was to understand the acceptability of the programme as well as to explore the barriers and enablers for making dietary changes. A qualitative descriptive approach to analysis was followed. In qualitative descriptive analysis, the researcher seeks to identify and learn about how people experience an event, or a process, or to learn about people’s perspectives, rather than to generate theory [31]. Focus groups transcripts were imported into NVivo-12 software for data management and coding. Data analysis was conducted using a thematic approach, guided by Braun and Clarke’s published guidelines [32]. Transcripts were first read by TR for familiarisation and then reread and coded. This was an inductive coding process where the codes were drawn from the raw information itself and the themes were predominantly data driven [33]. To establish credibility and confirmability of the emergent topics, coding was performed by a second researcher (AG) who was not involved in data collection. Topics identified by both TR and AG were reviewed, and in collaboration, related topics were collated to generate initial themes. Initial themes were then further refined, ensuring clear and encompassing definitions were generated when naming final themes and subthemes.

## Results

### Demographics

21 participants (20 females, 1 male), aged 23–77 years (mean 47.5, SD 12.3) took part in the online focus groups (MedDiet: *n* = 11; HEG: *n* = 10). Most participants (90.5%) were born in Ireland and education ranged from incomplete schooling at second level to master’s degree. A total of four focus groups were conducted; two with MedDiet participants and two with HEG participants. Each focus group consisted of 5–6 participants and lasted approximately sixty minutes. Participant characteristics are presented in Table 2.

**Table 2:** Participants characteristics

Characteristics (% or mean ± SD)	MedDiet group (*n* = 11)	HEG group (*n* = 10)	Overall group (*n* = 21)
Age (years)	47.5 ± 14.5	47.3 ± 10.2	47.5 ± 12.3
Female, *n* (%)	11 (100)	9 (90)	20 (95.2)
Born in Ireland, *n* (%)	9 (81.9)	10 (100)	19 (90.5)
Education, *n* (%)			
Secondary school Certificate/diploma Bachelor’s degree Master’s degree	1 (9.09)	0 (0)	1 (4.76)
	1 (9.0.9)	3 (30)	4 (19.04)
	5 (45.45)	3 (30)	8 (38.09)
	4 (36.36)	4 (40)	8 (38.09)
Currently employed, *n* (%)	8 (72.8)	10 (100)	18 (85.8)

The thematic analysis resulted in the generation of four key themes relating to the participants’ experience and perceptions.

### Theme 1: Motivation to join the programme

Participants reported different reasons for their motivation to take part in the MEDRA trial. Among the most frequently stated motivations were:

*Drug alternative* The majority of participants expressed a desire to find an alternative to their conventional drug treatment as a primary motivation for joining the study.

*“I've been looking for an alternative to the drugs” (HEG participant 3)*

*“Anything I can do to reduce any kind of drugs; I’ll give it a go” (HEG participant 7)*

*The notion of eating healthy for wellness* Most participants reported that the main reason for joining this study was that they believed that eating a healthy diet will help them feel better. Participants in both groups seemed eager to improve their symptoms and were hoping to feel better as a result of the programme.

*“I think changing my eating habits will make me feel better.” (HEG participant 1)*

*Evidence-based research study* A small number of participants expressed an interest in the MEDRA trial given it is a self-directed evidence-based research study about disease management.

*“It appealed to me that it was a scientific study, you know, and I just felt it was backed with evidence.” (MedDiet participant 9)*

*Lived experience of the MedDiet* Participants in both diet groups expressed a keen interest in trying the MedDiet. The MedDiet was perceived as something ‘different’ and ‘exotic’. There was some discouragement and disappointment expressed from two participants in the HEG group who indicated that they would have preferred to try something ‘new’.

*“I studied human nutrition so I knew a lot about the Mediterranean diet already and I just knew it would have beneficial effects for my body anyways, so that encouraged me to do it.” (MedDiet participant 4)*

*“… I really wanted to be in the Mediterranean diet group, it’s just something different, something exotic to try you know…” (HEG participant 1)*

### Theme 2. Benefits of the programme

Benefits of the dietary programme emerged as a key theme throughout the focus groups in both, the MedDiet and HEG group. In both groups, participants discussed how improving their diet quality resulted in a wide range of benefits. Reported benefits were grouped into four categories: symptom management, physical benefits, increased energy and strength and increased nutrition awareness.

*Symptom management* Participants in both diet groups described how they experienced less pain and morning stiffness by improving the quality of their diet. The majority of participants explained how they were able to reduce the amount or frequency of medications they were taking without experiencing any worsening in their symptoms. One participant reported that the dietary modifications helped her to refrain from using painkillers.

*“I didn't have to take any painkillers and my joints didn't swell as much as they usually do.” (MedDiet participant 2)*

*“You know in winter I would normally have to go on steroids for a couple of weeks or whatever, but I didn't need to do that.” (MedDiet participant 6)*

*Physical benefits* Improvements in skin and body weight were also reported by participants from both diet groups.

*“I felt my skin had improved, not that I had a problem with my skin, but I think I notice that about my skin.” (MedDiet participant 6)*

*“This has helped me lose a bit of weight as well and my husband noticed it.” (HEG participant 5)*

*Increased energy and strength* Participants perceived the dietary intervention as beneficial as it improved their energy levels and increased their strength and mobility. Two participants from the MedDiet group stated their participation in the programme has given them the ability to exercise more and carry heavier weights at the gym.

*“I started going to the gym three times a week.” (MedDiet participant 3)*

*“I noticed that my energy levels were much better than before, and I was lifting more weight at the gym.” (MedDiet participant 1)*

*Increased awareness of nutrition* Participants were impressed by how their nutritional knowledge improved towards the end of the intervention. They were happy to learn about portion sizes and became more aware of the types of food they were consuming. Participating in the programme helped them acquire a routine for keeping account of their food intake and develop a sense of self-monitoring.

*“It definitely helped me with like portion sizes and stuff that you know I never really considered before.” (HEG participant 8)*

### Theme 3. Factors influencing adherence to the dietary prescription

Participants reported challenges that prevented them from changing their dietary behaviours as well as the factors that encouraged and facilitated the adoption of healthy eating habits.

*Pain* The chronic pain experienced by participants was frequently described as a barrier to changing diet and especially adopting a healthy eating routine. Participants described pain as a barrier to adopting a healthier dietary pattern. One participant explained that whenever they were in pain, they would rely heavily on convenience foods and foods that are easy to prepare. Another participant stated that their chronic pain often leads to poor appetite and inadequate intake.

*“Whenever I felt sort of under the weather, I found myself just not being able to eat healthy, uhm, like I was always looking for a bag of crisps you know, all sorts of*

*comfort food.” (HEG participant 3)*

*“I can’t even imagine myself eating healthy when I’ve a flareup.” (MedDiet participant 10)*

***Accessibility*** Food accessibility was reported as a barrier among many participants in both diet groups but predominantly among individuals who were randomised to the MedDiet. Participants stated that, in Ireland, fresh fruits and vegetables consistent with the MedDiet are not always readily accessible. Moreover, food outlets serving Mediterranean-type meals were also scarce. Furthermore, participants discussed how the social restrictions to mitigate the risk of COVID-19 infection reduced the accessibility to certain food products, for example, one participant stated that they struggled to access low-fat products. A small number of participants who lived in the countryside indicated that it was challenging to access healthy foods or products consistent with the MedDiet in their local shops.

*“Well restaurants, for example. You know you might not have as big choice as you'd like in restaurants.” (MedDiet participant 3)*

*“.. I think like some grocery stores in the countryside, they don't have anything suitable for the Mediterranean diet or say the house is getting takeaway, the restaurants won’t have Mediterranean type meals.” (MedDiet participant 1)*

*Time* Time was perceived as both a barrier and enabler. This factor was mainly related to the time needed to plan and prepare healthy meals. Participants explained how their normal busy schedules prevent them from eating healthily. Many participants reported that COVID-19 related lockdowns and restrictions had given them a lot of extra time for cooking their own healthy meals at home. Planning and preparing meals and snacks in advance were considered key enablers to adopting a healthy diet.

*“The planning was time consuming I found in the beginning just to try and figure out what meals we were going to have but then I started preparing everything on a Sunday.” (MedDiet participant 7)*

**Taste***.* The acceptability of the MedDiet was enhanced by the taste and appearance of its constituents.

*“I don't think I was expecting all that olive oil but one benefit was that the olive oil tastes nice.” (MedDiet participant 4)*

*“I'm converting my husband as well. Yeah, you know, he's really happy to eat like that ... I mean, it's so tasty.” (MedDiet participant 2)*

*Social influences* The social environment including friends, family, peers was perceived as both a barrier and an enabler. Participants reported that it was easier for them to follow a healthy diet when they felt supported by others around them. Having an unsupportive social environment was often described as a barrier to eating a healthy diet. A number of participants explained how they occasionally felt pressured to eat more when they were around their families and how peer influence, while often coming from a good place, may encourage unhealthy eating behaviours.

*“The only real barrier I would say is incorporating the family.” (HEG Participant 7)*

*“It was much easier for me when my husband was enjoying the same food.” (MedDiet participant 5)*

*Knowledge/skills* Knowledge was identified as both a barrier and an enabler. Overall, participants showed great interest in obtaining information about diet for managing their condition and responded very positively to the resources that were provided to them. One participant illustrated how knowledge plays a role by giving an example about the different ways one can cook potatoes. He explained that, while there is nothing wrong with buying a bag of potatoes, what really matters is how the potatoes will be prepared, and this comes down to an individual’s level of knowledge. Moreover, lacking cooking skills was also perceived as a barrier by two participants who highlighted a need for teaching how to cook at an early age.

*“I had heard about the food pyramid before but didn’t know much about it. I felt like having the resources and all the recipes provided helped me in many ways.” (HEG participant 8)*

*“I suppose it's really the basic cooking skills. Yeah, it's lacking, and I suppose the other thing is education, just learning about the diet and the portion sizes and how to cook.” (MedDiet participant 9)*

*Food culture* The food culture was reported as a barrier by some participants, mainly those in the MedDiet group. Participants explained how the food culture in Ireland centres predominately on red meat, dairy and bread, and is at odds with the principles of the MedDiet.

*“I never realised how much I loved red meat, so cutting down on the red meat was that was difficult.” (MedDiet participant 2)*

*I think it's just that we’re like always eating sandwiches or bread and butter that kind of thing you know.” (MedDiet participant 10)*

*Cost* Many participants illustrated how the cost of either diet seemed to be prohibitive. One participant perceived cost as an enabler one and explained how spending a little extra on the healthier food options will ultimately save them the money they could be spending on clinic visits and RA medications.

*“I’ve had very few doctors’ visits over the past few months. I felt I saved that way so.*

*If the diet works for you, even though it might be you're spending it in the supermarket. You might be spending less at the doctor possibly or you know buying stuff to relieve your symptoms.” (HEG participant 7)*

*“I’d say it's a little bit more expensive to follow a Mediterranean diet…” (MedDiet participant 4)*

*Guidance/support* The RD’s supporting role was perceived as a valuable feature of the MEDRA trial. Participants recognised the potential benefit of regular follow up and guidance offered by the study RD. Participants in both groups explained how the individualised assistance and goal setting has given them a sense of security and accountability and made it easier for them to follow the diet. They felt supported throughout the study period and found that the RD took time to listen to them during the teleconsultations.

*“.. I felt like I could tell her things I wouldn’t normally talk about in clinic...” (HEG participant 2)*

*“I liked the fact that we were setting goals every couple of weeks, it made me feel sort of accountable you know and made it easier to stay on track” (HEG participant 1)*

*COVID-19* The health pandemic was regarded as both a barrier and an enabler. The restricted access to social outings and drink outlets was a huge enabler that was highlighted by almost all participants. However, several participants explained how the pandemic-related restrictions limited their ability to exercise.

*“I was more in control of my own diet, not traveling or eating in other people’s homes” (HEG participant 3)*

*“I couldn’t go for a swim or a long walk like I normally would.” (MedDiet participant 9)*

*‘Pick and Mix’* Participant discussed how the idea of being able to merge key elements of the MedDiet with a standard healthy diet would enable them to follow a healthy diet long term.

*“I will take some things from the Mediterranean diet on board for sure...” (MedDiet participant 8)*

*“I suppose it would be no harm to do the odd snacking on nuts so I'll keep doing that.*

*Yeah, and the fish. Obviously, the fish. I love the fish.” (MedDiet participant 3)*

*Doubts about effectiveness* While many people with RA believe that diet plays a critical role in their disease progression [34], a small number of participants expressed some hesitancy toward the role of diet in their condition. Although participants who experienced positive effects acknowledged that their participation in the dietary programme could have helped, they did not think diet alone was responsible for the major benefits:

*“.. it could be the new treatment I’m on and not the diet ...” (MedDiet participant 9)*

Another participant expressed interest in long term impact of the intervention on their disease.

*“Uhm, well again I'm on two new different medications now, I feel much better, but I don't know whether it was, you know, the drugs or the diet. I feel I would need to look at it over. It may be a long term longer term. I would be interested to see how I feel.*

*You know, over a period of a year or over six months or something” (HEG participant 6)*

### Theme 4. Advantages and disadvantages of telehealth

Participants discussed the advantages and disadvantages of the intervention being delivered through telehealth methods. In general, all participants were very positive about the mode of delivery, which they found very easy to work with. The teleconsultations via the video were experienced as more focused and less stressful than visiting the outpatient clinic. Most participants found the telehealth experience flexible and comfortable. In most cases, it was preferred over the need to go to the hospital specifically during COVID-19 pandemic. Many participants stated that they would never have participated in the programme if it was not delivered online mainly due to their other commitments and scarcity in time.

*Flexibility* Participants noted that the flexibility around call scheduling and the length of calls did not disrupt their busy schedules. They regarded the intervention as flexible and ‘fitting’ into daily life. Teleconsultations were described as very easy to integrate into daily lives. In addition, participants valued the ability to complete the online questionnaires in their own time.

*“You know it was flexible I must say. I could just fill the questionnaires at night when the kids are in bed and we would have the call during my lunch break at work.” (HEG participant 4)*

*“It’s the best option for me, it’s very flexible.” (MedDiet participant 10)*

*Safety* The video consultations were perceived as a safer option to in-person appointments. Given that people with RA are often at increased risk of infections due to immunosuppressive drugs [35], participants reported that they would be worried about picking up an infection if they had to visit the clinic during the pandemic.

*“It was definitely safer than having to go into the hospital during covid” (HEG participant 7)*

*Resource-saving* Resource-saving was identified as the most cited advantage to telehealth. Participants appreciated that they could access the telehealth intervention without having to travel, navigate public transport or pay for parking. Participants described the visits to their rheumatologists as very stressful and tiring because of the often-long transport times and the problems of finding a parking space close to the clinic.

*“I wouldn’t have been able to take part in this study if it wasn’t online, I would have to travel and that is time consuming.” (HEG participant 1)*

*Inability to perform physical measurements* The inability to perform body composition and anthropometric measurements was reported as a barrier to telehealth by one participant. However, most participants agreed that, for the purpose of this study, there was no need for in-person clinic visits.

*“I would have like say want to have some measurements done.” (MedDiet participant 5)*

*Absence of peer support* Absence of peer support was a major disadvantage discussed by participants in both diet groups. Participants highlighted how the mode of delivery prevented them from meeting others taking part in the programme leading to a feeling of isolation.

*“You know we could not meet others who were also on the programme and see how they were getting on” (MedDiet participant 2)*

## Discussion

Understanding participants’ experiences and key factors influencing the adoption of a new dietary pattern or improving diet quality is crucial for informing interventions to increase adherence and improve the implementation and delivery of dietary interventions. This qualitative study arm of the MEDRA study aimed to explore the experiences of participants following the completion of a telehealth-delivered intervention trial that aimed to assess the effects of improving diet quality through the adoption of a MedDiet or HEG in people with RA. Using semi-structured focus groups,the study also explored the barriers and enablers toward making and sustaining dietary change. The results revealed that the telehealth trial was well received, with participants reporting positive experiences and outcomes. Participants across both diet groups reported that they enjoyed taking part in the intervention and that they experienced several benefits as a result of improving their eating habits. Despite the substantial differences between the two dietary prescriptions, both interventions were viewed similarly by the participants with common themes identified across all four focus groups. The results from this study give insight into the potential beneficial role of telehealth in facilitating interventions and help indicate which aspects of the MEDRA trial were considered most effective. The findings also highlight fundamental issues to be addressed in future dietary interventions for this population.

Participants stated several reasons for deciding to join the MEDRA trial. The most common reason was their desire to find an alternative to their traditional drug treatment. This is consistent with several studies highlighting that people with RA frequently seek complementary and adjunctive therapies due to often severe side effects they experience from taking the drugs [36]. Consistent with the broader literature [37], potential health improvements and expected benefits from eating a healthy diet were regarded as other key motivators for taking part in the programme. In addition, most participants agreed that being involved in an evidence-based and facilitated study provides an opportunity to learn more about disease self-management. Interestingly, a number of participants stated that their involvement in the study was motivated by their interest in the MedDiet which was perceived as ‘new’ and ‘different’. This was further emphasised when participants from the HEG group spoke of their disappointment at not being allocated to the MedDiet group. This finding is in line with other qualitative studies in non-Mediterranean countries reporting that most participants not allocated to the MedDiet intervention felt disappointment about their group allocation [27, 38]. This finding also suggests that, whilst the MedDiet is becoming increasingly popular [39], there is a pressing need to promote the national Healthy Eating Guidelines in Ireland given that these are evidence- based recommendations with established health benefits and particularly in light of the low rates of compliance to these guidelines in Ireland [40].

Consistent with the broader literature on barriers to adopting a healthy eating pattern, the main barriers reported in this study were lack of time, perceived cost of healthy foods, lack of social support, food inaccessibility, and limited knowledge [41, 42]. Specific to a RA population, the chronic nature of pain experienced was highlighted as a major barrier to healthy eating. Participants explained how the pain experienced restricts their mobility and impedes their ability to form healthy habits, a result in line with another qualitative study with people with arthritis [42]. Therefore, tailored approaches that are simple to prepare and the ability to plan ahead of time when feeling better may be important for this population. Participants also identified several enabling factors that influenced their intention to change their current eating habits. While there is no evidence on factors influencing dietary change in a RA population, several studies have reported on determinants of change for other lifestyle behaviours including physical activity [43, 44]. Proposed strategies included education and guidance, both of which were identified as enabling factors by participants in this study. Contrary to studies that found a direct association between COVID-19 and overeating [45, 46], participants in the present study found that the home confinement due to the pandemic provided them with extra free time to prepare healthy foods. Furthermore, knowledge was highlighted as both a barrier and an enabler. Participants in both groups highlighted the importance of education and expressed receiving very limited dietary advice from their healthcare providers. Very little is known about the extent of dietetic involvement in rheumatology teams in Ireland or elsewhere [47]. In addition to the potential dietary benefits on RA itself, people with RA have a high burden of comorbidities for which diet is beneficial [48], therefore, potential benefits of improving diet quality must be clearly communicated within this population. Access to nutrition counselling, clear dietetic advice and qualified dietitians who can provide individualised guidance regarding beneficial dietary changes must be easily available. In the presence of no cure for RA [49], the role of a multidisciplinary team is extremely important and dietetic involvement, even in a virtual format, as demonstrated by the study herein, is warranted.

Another topic that emerged was the ‘pick and mix’ approach, participants explained how being able to incorporate certain components of the MedDiet into a standard healthy Irish diet is easier than switching completely into a typical MedDiet. Moreover, the MedDiet is a dietary pattern that is also palatable [50], participants from the MedDiet group indicated that they enjoyed the MedDiet and with a strong emphasis on the taste and flavour of the MedDiet foods. This suggests that the MedDiet, with its specific constituents, such as high amounts of healthy fats, is well-accepted in an Irish population with RA despite the national food-based dietary guidelines being based on low-fat foods.

One distinction between the MEDRA trial and other dietary interventions in RA is the method of delivery. In light of COVID-19 restrictions and public health measures, telehealth methods were used for the dietary intervention in the MEDRA trial [23]. While most participants in our study did not have any experience with telehealth prior to the study, the overall finding was that people with RA generally had positive perceptions about the telehealth component of the intervention and perceived the new mode of delivery as flexible, safe and resource-saving. The flexibility around call scheduling was greatly valued by all the participants. These findings are in line with another study describing the perceptions of people with RA toward telehealth [21]. Nevertheless, the main disadvantage reported by participants was the absence of peer support and inability to meet others who were taking part in the programme. This was a major disadvantage reported in many qualitative studies on telehealth with participants expressing interest in sharing their experiences with other participants on the programme [51, 52]. Overall, our data suggests that telehealth may provide a well-accepted approach for remotely delivering dietetic services to support disease self- management in people with RA. In this context, it is important to note that, for individuals with limited access to technology or inadequate technological literacy, face-to-face service delivery remains essential. Therefore, a hybrid approach which uses a combination of telehealth, and in-person care may be the best option. Future interventions must also consider including virtual support group sessions for participants, an option that has not been explored yet.

### Strengths and limitations

A comprehensive search of present literature yielded no prior research investigating the experiences and perspectives of dietary intervention in people with RA, making this study the first of its kind. To the authors’ best knowledge, this is also the first study to offer qualitative insight into the barriers and enablers to following a Mediterranean dietary pattern in Ireland. The results of this study provide a foundation of understanding to guide the development of effective patient-centred dietary interventions for people with rheumatic conditions and can also be used in the development of MedDiet-based interventions in non- Mediterranean countries. Understanding factors that influence dietary change will help create targeted approaches to maximise dietary change in a RA population. Capitalizing on the identified enablers such as knowledge and guidance, may lead to increased participation in future dietary interventions.

The focus group sessions were moderated by an independent researcher who had no prior contact with participants and used the same semi-structured question guide to ensure consistency. A wide age range capturing varying views and perspectives was included in the study. Data analysis was carried out by two independent researchers to ensure results were not based exclusively on a single and perhaps biased interpretation. Methodological rigour was addressed using numerous strategies including: adherence to the 15-point checklist of criteria for good thematic analysis [53] and ongoing documentation of the researcher’s personal reflections relating to her values, beliefs, previous experience as a dietitian in order ensure reflexivity [54]. Moreover, the recently published assessment tool by Braun and Clarke (2021) was used to guide the study and evaluate the quality of this research [55].

However, the study has a number of limitations to consider. Firstly, the intervention was delivered online so it is possible that responses are not fully representative of the Irish population with RA since individuals with limited or no access to technology may have different views on telehealth compared to those who regularly use it. In addition, participants were recruited mainly though the patient organisation ‘Arthritis Ireland’s’ social media platforms, it is possible that the participants in this study may be highly motivated with greater self-efficacy and interest in their health. As such, the results may not accurately reflect views of the wider population with RA. In addition, the responses regarding telehealth may be affected by the necessity of this modality during the COVID-19 pandemic so it would be interesting to repeat same questions post-pandemic and to determine current perspectives on teleconsultations. It is important to mention that the focus groups reflected on a short period of temporary diet changes so it remains unknown whether the positive experiences will last in the long run. Limitations associated with the small sample size must also be acknowledged while recognizing that the primary aim of this qualitative research was not centred around generalizability. Instead, the purpose of this research was to explore and understand participants’ experience and perceptions following participation in a telehealth-delivered dietary intervention. Thus, while the small sample size may limit the external validity of the findings, it does not undermine the study’s intended aim of generating detailed insights into participants’ experiences. In addition to the small sample size, only English-speaking participants were included in the study, therefore, our findings may not fully represent the experiences and perspectives of individuals who do not speak English. Moreover, most participants in this study were highly educated with a university degree so the sample findings may not be representative of the wider population or individuals with other educational or socioeconomic backgrounds. Future research exploring males’ experiences is also warranted given that the sample included in our study comprised primarily of females.

## Conclusion

In conclusion, the themes generated from this qualitative analysis showed that participants had overall positive experiences in both the MedDiet and HEG interventions. This was the first study to demonstrate that a MedDiet was accepted in a non-Mediterranean Irish population. The study also demonstrated that a dietary intervention programme delivered by a RD through telehealth methods is an acceptable complement to face-to-face care in adults with RA. Participants' motivations for taking part and perceived benefits highlight that participation in dietary studies is perceived as important and can produce benefits in terms of physical and psychological well-being. In addition, the data indicates that adults with RA are often interested in dietary programmes which provide evidence-based and expert input. These findings may facilitate research translation to practice and contribute toward a more wholistic approach for RA management. Lastly, the identified themes including barriers and enablers to adopting healthier eating patterns should be addressed in the development of future interventions to effectively promote and facilitate dietary adherence.

## Data Availability

Raw data that support the findings of this study are available from the corresponding author, upon reasonable request.
